# Performance Analysis of *Anaplasma* Antibody Competitive ELISA Using the ROC Curve for Screening of Anaplasmosis in Camel Populations in Egypt

**DOI:** 10.3390/pathogens9030165

**Published:** 2020-02-27

**Authors:** Omid Parvizi, Hosny El-Adawy, Uwe Roesler, Heinrich Neubauer, Katja Mertens-Scholz

**Affiliations:** 1 Institute of Bacterial Infections and Zoonoses, Friedrich-Loeffler-Institut (Federal research institute for Animal Health), 07743 Jena, Germany; hosny.eladawy@fli.de (H.E.-A.); Heinrich.Neubauer@fli.de (H.N.); Katja.Mertens-Scholz@fli.de (K.M.-S.); 2 Faculty of Veterinary Medicine, Kafrelsheikh University, Kafr El-Sheikh 33516, Egypt; 3 Institute for Animal Hygiene and Environmental Health, Free University Berlin, 14163 Berlin, Germany; Uwe.Roesler@fu-berlin.de

**Keywords:** anaplasmosis, camel, ROC curve, real time PCR, cELISA

## Abstract

Anaplasmosis is a tick-born and potential zoonotic disease caused by *Anaplasma* (*A.*) *phagocytophilum*, *A. ovis*, *A. platys* and *A. capra*. *Anaplasma marginale* affecting bovines and camels causing significant economic losses. Camels as an integral part of the socio-economic lifestyle of nomads in semi-arid to arid ecosystems are prone to suffer from subclinical *Anaplasma* infections. This study aimed to determine the performance and adaptation of commercial competitive *Anaplasma* ELISA (cELISA) as a tool for screening the seroprevalence of anaplasmosis whitin the camel populations in Egypt. This study was based on the serological investigation of 437 camel sera collected between 2015 and 2016 during a Q fever prevalence study in Egypt using commercially available cELISA for the detection of antibodies specific for *Anaplasma* in bovine serum. The receiver operating characteristic (ROC) curve, an analysis method for optimizing cutoff values in cELISAs, was used to estimate the sensitivity and specificity using 76 true as serological positive (*n* = 7) and negative (*n* = 60) for *Anaplasma* antibodies. ROC curve analysis was done for 7 true positive and 60 true negative bovine samples and 7 true positive and 29 true negative camel samples serum. Real time PCR and/or conventional PCR was applied to confirm *Anaplasma* spp. specific-DNA in camel serum as an indication of a true positive and true negative for ROC analysis. Chi square analysis was performed to estimate the association between risk factors and anaplasmosis in camels. The cutoff value was determined as 0.42 (*p* value ≤ 0.001). Data simulation with randomly generated values revealed a cutoff value of 0.417 (*p* ≤ 0.001) with resulting 58.1% *Se* and 97.8% *Sp*. Seven true positive and 29 true negative camel serum samples was confirmed by PCR. Using the estimated cut off, the seroprevalence in the Nile Valley and Delta and the Eastern Desert domain was 47.4% and 46.4%, respectively. The potential risk factors as domains and origin of animals were less significantly associated with the prevalence of anaplasmosis (domains: χ(2) = 41.8, *p* value ≤ 0.001 and origin: χ(2) = 42.56, *p* value ≤ 0.001). Raising awareness especially for veterinarians and animal owners will significantly contribute to the best understanding of anaplasmosis in camels in Egypt. Alternative (*in silico*) validation techniques and preliminary prevalence studies are mandatory towards the control of neglected anaplasmosis in the camel population.

## 1. Introduction

Camels are utilized for milk, meat, wool and hide production as well as for transport since 4000 BC [1]. Most camel populations are kept in India and at the Horn of Africa [1]. In Egypt, the camel population has steadily increased between 2002 and 2015 [2].

*Anaplasma* and *Ehrlichia* are obligate intracellular alphaproteobacteria and belonging to order Rickettsiales, family Anaplasmataceae that are transmitted to vertebrate hosts by ticks of the family Ixodidae and cause symptoms similar to febrile diseases in humans and domestic animals like the camel [3,4]. Anaplasmosis often occurs in animals of tropical and subtropical regions but also in North America, Europe and the Mediterranean region [3,5]. Anaplasmosis can be transmitted mechanically by ticks, tabanid vectors, iatrogenically and transplacentally [5]. Anaplasmosis usually manifests as a subclinical infection or as co-infection in camels [6]. El-Naga and Barghash, 2016 reported clinical cases with fever, enlarged lymph nodes, anemia and jaundice in camels [7]. Other studies and deposited sequences (NCBI) indicated the presence of *Anaplasma camelii*, *A. marginale*, *A. centrale*, *A. ovis* and *A. platy* DNA in camels [8].

Routine diagnosis of anaplasmosis in camels is based on clinical signs and microscopic examination of blood samples. Proper selection of currently available diagnostic assays to obtain the maximal confirmation potential was dependent upon recording the detailed clinical history that identifies the time interval from the onset of symptoms appearance to the investigation of the clinical specimens [9].

Although the indirect fluorescent antibody technique (IFAT) is one of the most commonly used tests, ELISA has more advantages over it, since results can be obtained directly through a microplate reader, which make it possible to evaluate a larger number of serum samples and avoiding problems with doubtful interpretations [10].

Real-time PCR assay is considered as a rapid, sensitive and accurate diagnostic adjunct when compared with direct blood smear analysis for the identification of anaplasmosis. Serologic detection correlates poorly with PCR or blood smear analysis and more accurately reflects the collective exposure history occurring from late in the acute infection period into convalescence [9].

Statistical approaches can significantly help amending the performance of analytical tests. Receiver operating characteristic (ROC) curve analysis [11] and a World Organisation for Animal Health (OIE) recommended tool [12] were commonly used to optimize the cutoff values in ELISAs to find the best correlation for sensitivity (*Se*) and specificity (*Sp*) [13,14,15,16]. Some other methods to estimate the cutoff values are (1) mean value plus three standard deviations of negative controls [17]; (2) Cutoff= $\bar{X}$neg + 0.13 $\bar{X}$pos where $\bar{X}$ is the mean [18,19] and (3) $Cutoff=\bar{X}+fSD$” with f=t1+(1n) [19,20]. These methods are based on values obtained with negative sera. Frey et al. (1998) relied on the upper tail of the *t*-distribution of negative samples [20].

Anaplasmosis has been reported in some parts of Egypt in cattle, buffaloes, camels and humans. Nevertheless, there is a lack of regular monitoring and countermeasure programs in the field. *Anaplasma marginale* is most often reported and confirmed in cattle, camels and arthropods from various host animal species. Anaplasmosis in camels was reported in Matrouh, South Sinai, Assuit and Luxor in Egypt. The diagnosis of anaplasmosis in Egypt was dependent on cELISA, IFA, microscopic examination and PCR [7,21,22,23,24,25,26,27,28,29].

A comprehensive prevalence study of camel anaplasmosis in Egypt and the adaptation of the commercial cELISA used for bovine to test camel sera are missing. Thus, this study aimed to adapt the commercial competitive ELISA (cELISA) used in bovines for camel sera and preliminary camel sera prevalence was analyzed.

## 2. Materials and Methods

### 2.1. Sampling and Serological Testing

Serum samples used in this study were originally collected between October 2015 and March 2016 in Egypt for a Q fever screening study in Egypt [30].

**Figure 1:**
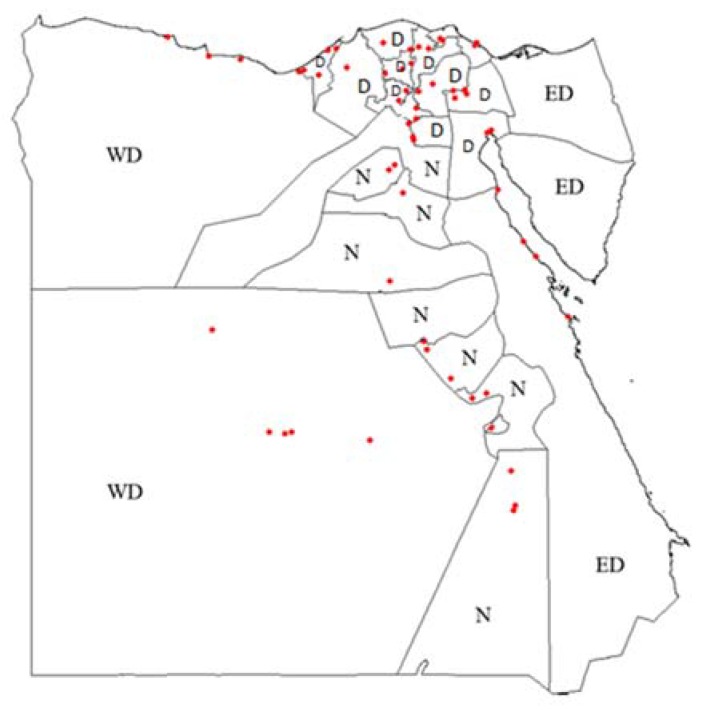
Geographical location of randomly selected sampling sites (red dots) in Egypt using GPS data. Delta (D), Nile Valley (N), Western Desert (WD) and Eastern Desert (ED).

In total, 437 camel sera were collected from 24 governorates in Egypt. There were no sample collected from Sinai, Assuit, and Minya. Governorates were assigned into three domains: the *Western Desert*, the *Eastern Desert* the *Nile Valley* and the *Delta* region (Figure 1).

Data including age (≤4 or >4 years), husbandry system (stable/stationary, pasture and nomadic) and tick infestation were recorded in Table 1.

Sera were screened for specific antibodies against *Anaplasma* spp. using a commercial competitive ELISA v2 (Veterinary Medical Research and Development Inc., Pullman, WA, USA) for the detection of antibodies specific for *Anaplasma* in bovine serum samples according to the manufacturer’s instruction. This assay had a sensitivity (98%) and specificity of 100% in bovines, which were calculated from data generated by diagnostic laboratory field testing [31].

Additionally, 67 cattle samples, previously tested as serological positive (*n* = 7) and negative (*n* = 60) for *Anaplasma* antibodies were included as positive and negative control serum. ROC was used to evaluate the prediction of sensitivity and specificity [32].

### 2.2. DNA Preparation and PCR Amplification

DNA was extracted from seropositive and seronegative serum samples using the High Pure PCR Template Preparation Kit (Roche, Mannheim, Germany) according to the manufacturer’s instructions. The concentration and quality analysis of DNA in each sample was measured using a Nano-drop1000^®^ (Thermo Fisher, Wilmington, NC, USA). DNA amplification was done using real time- and/or conventional PCR.

The real time TaqMan^TM^ PCR was performed using the AmpliTest *Anaplasma*/*Ehrlichia* spp. Kit (Amplicon Ltd., Wrocław, Poland) for quantitative detection of *Anaplasma* DNA according to the manufacturer’s guidelines. The result of the cycle threshold (Ct) value ≤38 was considered ‘positive’ and samples had a Ct value between 38 and 40 were considered ‘suspected’.

Conventional PCR was performed as described previously [32]. The PCR reaction was done using a Phusion Flash High-Fidelity PCR Master Mix (Thermo Fisher, Darmstadt, Germany) and primers MSP-5 254 F: 5′-GCA TAG CCT CCG CGT CTT TC-3′ and MSP-5 779R: 5′-ACA CGA AAC TGT ACC ACT GCC-3′ to amplify a 525 bp fragment of the major surface protein (MSP5) gene

### 2.3. Performed ROC Analyses

Diagnostic specificity, sensitivity and predictive values were determined by receiver operating characteristic (ROC) analysis (MedCalc statistical software, version 9.3.0.0). Based on the optical density (OD) values of the cELISA, positive and negative results ROC can be generated. Usually these data are good coverage, which means that all values are within the control range.

(1) Control interval=mean of Pos./Neg control±3∗standard deviation

(2) $$\begin{array}{l} True\;pos.baseline\\ =[(mean\;of\;pos.control-2\\ {}*standard\;deviation\;(smallest\;OD\;value),mean\;of\;pos.control+2\\ {}*standard\;deviation\;(greatest\;OD\;value)] \end{array}$$

(3) $$\begin{array}{l} True\;neg.baseline\\ =[(mean\;of\;neg.control-2\\ {}*standard\;deviation\;(smallest\;OD\;value),mean\;of\;neg.control+2\\ {}*standard\;deviation\;(greatest\;OD\;value)] \end{array}$$

A true positive and negative baseline established the probabilities of positivity or negativity were calculated to determine the upper/lower margin (limit) of the distribution of the control sera. The sera with the closest values to this limit can be selected as the true positive and negative range, due to the highest probability of positivity/negativity for further analyses.

ROC curve analysis was done for 7 true positive and 60 true negative bovine samples and 7 true positive and 29 true negative camel serum using SPSS Statistics software^®^ (Armonk, IBM Corp, USA, version 19) to obtain Ct, Se and Sp values. These values were used to determine seroprevalence of 347 camel sera. In addition, the above formula was used for screened camel sera, baseline values were obtained true positive and true negative data for using in simulation analysis. In the simulation analysis of the 2300 field serum samples, random data (true negative = 2000 and true positive = 300) were generated using the positivity and negativity area of each plate.

ROC analysis for data reconstruction was done with 10% expected error. It should be noticed that wells with an optical density ≤0.20 were uncolored when inspected visually to assure a higher probability of positivity. In addition, for this study true positive/true negative samples were confirmed with real time PCR and/or conventional PCR with the exception of a true negative of bovine. These were selected from a true negative baseline.

### 2.4. Statistical Analyses

The metadata of collected serum in this study were categorized in age (≤4 and >4 years), tick infestation and the animals husbandry system (stable/nomadic). A chi-square or Fisher’s exact test was used to determine the association of the disease with these risk factors. Seroprevalences were calculated as the proportion of positive results in a population. 

## 3. Results

Seven true positive and 29 true negative camel serum samples were confirmed by real time PCR as an indication of the true positive and true negative for ROC analysis.

**Figure 2:**
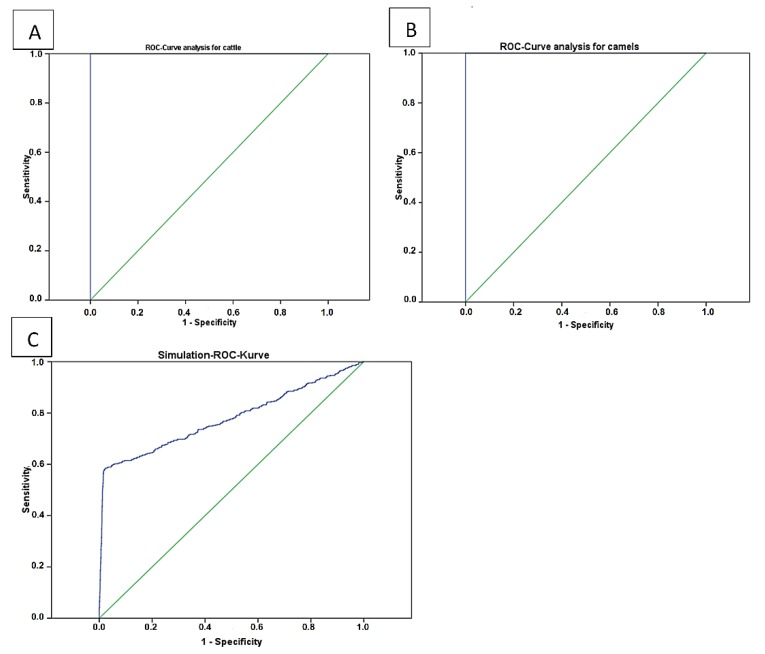
Display of the performance analysis of the cELISA Anaplasma kit V2 using true positive and true negative samples. Both analyses showed 100% *Se* and *Sp* ((**A**) cattle and (**B**) camels). A simulation (**C**) was done with 2300 randomly generated data involved positives (300) or negatives (2000). This data contain a 10% intentional error.

The results of statistical analyses for threshold optimization of the cELISA V2 for use in cattle (Figure 2A) and camel (Figure 2B) sera are shown in Table 2 and Figure 2. These values were 0.42 (*p* ≤ 0.001) in camels and 0.4022 (*p* ≤ 0.001) in cattle. 

**Figure 3:**
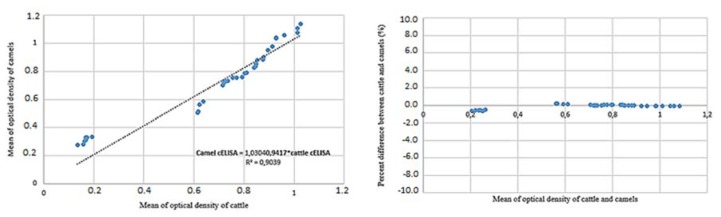
A scatter plot of values of the cELISA Anaplasma kit V2 in camel vs. cattle sera that shows good correlation between two tests. This good agreement favors the use in camels. The percent difference between the analysis of cattle and camel sera is showed the true extent of the bias of optical density (OD). This means that in this case the number of infected animals may be a little bit less/greater than in reality.

A scatter plot of the mean optical density from cattle sera values vs. the sera of camels showed a correlated relationship (Figure 3). Percent differences vs. mean results of cattle and camel sera provided average discrepancy reported error estimates and true errors, which shows the true extend of the bias at a low optical density (Figure 3) [33,34]. This analysis proved good correlation between two tests in cattle and camel serum.

Data simulation with randomly generated values revealed a cutoff value of 0.417 (*p* ≤ 0.001) with resulting 58.1% *Se* and 97.8% *Sp*. 

The overall seroprevalence of anaplasmosis in camels (34.1%) was detected after optimization of the cELISA cutoff (Ct = 0.42). Nile Valley and Delta and Eastern Desert domains showed 47.4% and 46.4% seroprevalences, respectively. Of the camels 95.7% that were kept nomadic showed 33.7% seroprevalence.

There was no significant associated between anaplasmosis and age, the husbandry system and tick infestation (Table 3). The overall rate of camels infested with ticks was 10.7%. Camels younger than 4 years were highly infected than older (41.2% vs. 32.1%). Domain and origin of animals were found to be less significant associated risk factors for camel anaplasmosis (Table 3).

The majority of seropositivity 77.4% (*n* = 31) was determined in Aswan governorate from Nile Valley and Delta followed by 46.4% (*n* = 69) in red sea from Eastern Desert (Table 4).

## 4. Discussion

Anaplasmosis is known in Egypt since 1966 in bovines and the presence of various species of *Anaplasma* were confirmed by the use of PCR in Egypt [7]. 

The descriptive and analytic epidemiological methods to describe the dynamics, prevalence and risk factors of infected populations through an improved process for data collection and plan for novel interventions helps to improve the understanding of the disease and its control [35,36]. 

The commercial *Anaplasma* cELISA V2 kit from Pullman, USA, has been previously validated for use in the diagnosis of *A. ovis* in sheep with 100% specificity (95% CI: 96.7–100%) and 100% sensitivity (95% CI: 95.7–100%) [15] and with 96.5% sensitivity and 98.1% specificity [16].

No commercial serological test available for the detection of anti-*Anaplasma* antibodies in camel serum. Thus, there was a clear need for first steps to adopt a bovine test kit for use in camels. This study was aimed to validate the commercially available cELISA for screening the anaplasmosis in camel serum. Subsequently this optimization test was used to estimate a preliminary prevalence of anaplasmosis in the Egyptian camel population.

Due to a lack of a sufficient pool of true negative and true positive sera, an in silico simulation for 2300 randomly generated data with 10% error has been done and resulted in 97.8% *Sp*. and 58.1% *Se*. The calculated lower sensitivity of the test in this study may have resulted from the included error for estimating the true positive and true negative range. In some test plates, few camel sera had a higher optical density than the optical density of the negative controls. This fact shifted the results of true positive/true negative to a higher error and to a reduced the test sensitivity. Other reasons may be caused by a different affinity of species-specific antibodies [33] of camels vs. those of bovines as well as the IgG deficiency of camels [37,38], which may explain the fluctuations of the area under the curve and the different Se values as shown in Figure 3C. Truly negative and positive controls will need and have a positive effect on future validations. In this study, 7 true positive and 29 true negative camel serum samples were confirmed by real time PCR as an indication of a true positive and true negative for ROC analysis.

Hence, ROC analysis as a traditionally risk prediction model has shown that this cELISA can be used to detect anti-*Anaplasma* antibodies in camel sera and to estimate the preliminary prevalence of anaplasmosis in camels. At present, it might already be used in early warning systems and to monitor changes in the activity of the disease. Considering the increasing importance of camels in the future it therefore makes sense to further validate the WMRD *Anaplasma* cELISA kit for use in camels. It has to be stressed that there does not exist other studies to compare these in silico findings. Simulation would have been more effective and realistic if data from other studies were available. Chi square analyses revealed that the domain and origin of animals are the only significant risk factor (domains: χ(2) = 41.8, *p* value ≤ 0.001 and origin: χ(2) = 42.56, *p* value ≤ 0.001). These may be due to the lack of a proper distribution of health policies in most of the areas and the origin of animals as a source of disease transmission through the importation. 

In this study, bovine serum and bovine controls serum provided with this commercial cELISAv2 kit confirms that cELISA can be used with confidence to determine %I and to confirm the presence or absence of anti-*Anaplasma* antibody in camel serum. The results of this study proved that cELISAv2 kit was validated for the detection of anti-*Anaplasma* antibody in camels. The cELISA used in this study appeared to meet the criteria for use in diagnosing anaplasmosis and screening in camels for the presence of the *Anaplasma*-specific antibody.

Alternative (*in silico*) validation techniques and preliminary prevalence studies are the first steps towards control of neglected anaplasmosis in the generally untended but increasingly important farm animal camel.

It can be assumed that raising of society awareness especially in veterinarians and animal owners will significantly contributed to our understanding of anaplasmosis in Egypt.

## Figures and Tables

**Table 1:** Number (%) of animals sampled per domain with age group, origin of animals, husbandry systems and number of camel infested with ticks.

Domain		Western Desert 193 (44.2%)	Nile Valley and Delta 175 (40%)	Eastern Desert 69 (15.8%)	Total Samples 437
Age	≤4 years	32 (16.6%)	48 (27.4%)	17 (24.6%)	97 (22.2%)
	>4 years	161 (83.4%)	127 (72.6%)	52 (75.4%)	340 (77.8%)
Origin (Egypt/other country)		193/0 (100%/0)	13/162 (7.4%, 92.6%)	0/69 (0/100%)	206/231 (47.1%/52.9%)
Husbandry	Stable	0	15 (8.6%)	0	15 (3.4%)
	Nomadic	193 (100%)	133 (76.0%)	69 (100%)	395 (90.4%)
	Missing	0	27 (15.4%)	0	27 (6.2%)
Tick infestation		0	13 (7.4%)	21 (10.0%)	34 (7.78%)

**Table 2:** Detailed data of receiver operating characteristic (ROC) analysis for cattle, camels and a simulation for camels.

Animal Species	Samples		Area Under the Curve					Coordinates of the Curve		
	Positive	Negative	Area	Std. Error	Asymptotic Signs	Asymptotic 95% Confidence Intervals				
						Low Bound	Upper Bound	Positive	Sensitivity	Specificity
Cattle	7	60	1.000	0.000 (<001)	0.000 (<001)	1	1	≤0.18	0.857	0 (100%)
								≤0.40 *	1 *	0 (100%) *
								≤0.61	1	0.017 (98.3%)
Camels	7	29	1.000	0.000 (<001)	0.000 (<001)	1	1	≤0.33	0.857	0 (100%)
								≤0.42 *	1 *	0 (100% ) *
								≤0.51	1	0.034 (96.6%)
Simulation for camels	470	1830	0.779	0.015	0.000 (<001)	0.750	0.807	≤0.42	0.581	0.021 (97.7%)
								≤0.42 *	0.581 *	0.022 (97.8%) *
								≤0.42	0.581	0.022 (97.8%)

* Cut off values, *Se* and *Sp*. The simulation data were randomly generated after the true positive/true negative baseline for each plate was predicted based on the formula in the Materials and Methods.

**Table 3:** Associated risk factors for anaplasmosis in camels in Egypt.

Risk Factors		cELISA			Chi-Quadrat-Pearson	Phi and Cramer Value
		No. of Positive Animals				
		Proportion in Total Positive Animals (%)		Proportion in Population (Seroprevalence)		
Domain	Western Desert	34	22.8	17.6	Χ(2) = 41.8 (*p* value ≤ 0.001)	0.309 (*p* value ≤ 0.001)
	Nile Valley and Delta	83	55.7	47.4		
	Eastern Desert	32	21.5	46.4		
	Total	149	100	34.1		
Origin (Egypt/other country)		39/110	26.2/72.5	18.9/48.6	Χ(2) = 42.568 (*p* value ≤ 0.001)	0.312 (*p* value ≤ 0.001)
Age group	≤4 years	40	22.2	41.2	Χ(1) = 2.899 (*p* value = 0.093)	0.080 (*p* value = 0.093)
	>4 years	109	77.8	32.1		
Husbandry	Stable	6	4.3	0.4	Χ(1) = 0.258 (*p* value = 0.61)	0.025 (*p* value = 0.611)
	Nomadic	133	95.7	33.7		
	missing	10	6.7	10/27 = 37		
Tick infestation		16	10.7	47.1	Χ(2) = 3.819 (*p* value = 0.148)	0.0930 (*p* value = 0.148)

**Table 4:** Seroprevalence of anaplasmosis in camels in different governorates using cELISA.

Domain	Governorate	No. of Tested Camels	Seroprevalence *n* (%)
**Western Desert Area**	Matrouh	91	12 (13.2%)
	New valley	102	22 (21.6%)
**Eastern Desert Area**	Red Sea	69	32 (46.4%)
**Nile valley and Delta Area**	Alexandria	8	1 (12.5%)
	Aswan	31	24 (77.4%)
	Beheira	8	2 (2.5.0%)
	Beni-Suef	10	5 (50.0%)
	Cairo	8	3 (37.5%)
	Dakahlia	8	3 (37.5%)
	Damietta	8	3 (37.5%)
	Fayoum	8	3 (37.5%)
	Gharbia	6	2 (33.3%)
	Giza	7	3 (42.9%)
	Ismailia	7	2 (28.6%)
	Kafr el-Sheikh	5	3 (60.0%)
	Luxor	9	6 (66.7%)
	Menofia	7	5 (71.4%)
	Port Said	8	3 (37.5%)
	Qena	11	4 (36.4%)
	Qualyubia	1	1 (100%)
	Sharkia	7	3 (42.9%)
	Sohag	10	5 (50.0%)
	Suez	8	2 (25.0%)
Total		437	149 (34.7%)
